# Molecular Modeling on Structure-Function Analysis of Human Progesterone Receptor Modulators

**DOI:** 10.3797/scipharm.1105-03

**Published:** 2011-06-30

**Authors:** Ria Pal, Md Ataul Islam, Tabassum Hossain, Achintya Saha

**Affiliations:** Department of Chemical Technology, University of Calcutta, 92, A.P.C. Road, Kolkata-700009, India

**Keywords:** Human progesterone receptor-A, Binding affinity, Quinoline and cyclocymopol monomethyl ether derivatives, QSAR, Pharmacophore mapping, Docking

## Abstract

Considering the significance of progesterone receptor (PR) modulators, the present study is explored to envisage the biophoric signals for binding to selective PR subtype-A using ligand-based quantitative structure activity relationship (QSAR) and pharmacophore space modeling studies on nonsteroidal substituted quinoline and cyclocymopol monomethyl ether derivatives. Consensus QSAR models (Training set (Tr): *n*_Tr_=100, *R^2^_pred_*=0.702; test set (Ts): *n*_Ts_=30, *R^2^_pred_*=0.705, *R^2^_m_*=0.635; validation set (Vs): *n*_Vs_=40, *R^2^_pred_*=0.715, *R^2^_m_*=0.680) suggest that molecular topology, atomic polarizability and electronegativity, atomic mass and van der Waals volume of the ligands have influence on the presence of functional atoms (F, Cl, N and O) and consequently contribute significant relations on ligand binding affinity. Receptor independent space modeling study (Tr: *n*_Tr_=26, *Q^2^*=0.927; Ts: *n*_Ts_=60, *R^2^_pred_*=0.613, *R^2^_m_*=0.545; Vs: *n*_Vs_=84, *R^2^_pred_*=0.611, *R^2^_m_*=0.507) indicates the importance of aromatic ring, hydrogen bond donor, molecular hydrophobicity and steric influence for receptor binding. The structure-function characterization is adjudged with the receptor-based docking study, explaining the significance of the mapped molecular attributes for ligand-receptor interaction in the catalytic cleft of PR-A.

## Introduction

Estrogen and progesterone are two prime female reproductive hormones, have effects on multiple organs beyond reproductive system. Their actions are mediated through receptor-based gene stimulation. The human progesterone receptor (hPR) is a member of the intracellular receptor (IR) superfamily that includes the human androgen (hAR), estrogen (hER), glucocorticoid (hGR) and mineralocorticoid (hMR) receptors. Two different isoforms, A and B of hPR are present in various target organs of progesterone [1]. It is observed that hPR-B acts mainly as progesterone-responsive gene activator, whereas hPR-A functions as modulator of hPR-B activity and repressor for other IRs, suggesting hPR-A to be an important modulator for steroid hormone receptor actions [2–5]. Primary uses of hPR agonist and antagonist combined with estrogen are for the purpose of birth control, hormone replacement therapy, endometriosis, dysfunctional uterine bleeding, dysmenorrhoea, endometrial cancer, uterine leiomyomas, breast cancer, meningiomas and others [6, 7].

**Fig. 1:**
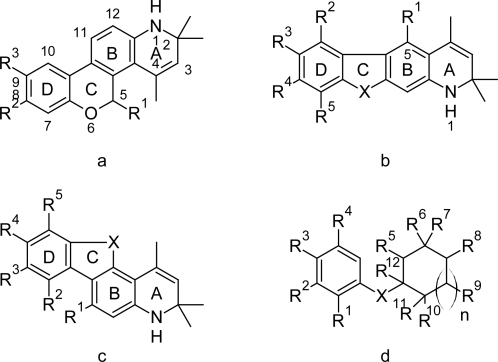
General structure of progesterone receptor modulators a: 1,2-dihydrochromeno[3,4-*f*]quinoline, b: 6-aryl-1,2-dihydro-2,2,4-trimethylquinolines (linear), c: 6-aryl-1,2-dihydro-2,2,4-trimethylquinolines (angular) and d: cyclocymopol monomethyl ether.

Focus on development of more selective and efficacious hPR modulators, including agonists and antagonists, have increased to a great extent considering the unwanted effects due to cross-reactivities with other IRs (hAR, hGR, hER, hMR) and GABA (γ-amino butyric acid) receptor [8, 9]. Binding affinity of nonsteroidal molecules in baculo-virus expressed hPR-A receptor analyses the interactions with the nuclear receptors to agonists, antagonists or partial agonists [9]. The nonsteroidal substituted quinoline derivatives [10] (Fig. 1a–1c) and cyclocymopol monomethyl ethers [7] (Fig. 1d) have been described for their interactions with PR subtype A as antagonists. The present work has been taken up to explore the essential chemical features of the molecular scaffold necessary for binding affinity to hPR-A using ligand-based molecular modeling techniques.

Molecular modeling is one of the cheminformatics techniques providing detailed information of a molecular system [11]. Computer aided drug designing (CADD) covers enormous fields ranging from pharmacophore mapping, multi-dimensional QSAR studies, receptor based approaches and binary screening to compound clustering. 3-D QSAR and docking studies of steroidal [12] and non-steroidal [13] analogs elucidate the binding interaction with PR. Docking experiment substantiated as a tool for discovery of pyrazoline-based antagonists [14, 15], non-steroidal agonists [16, 17], and steroidal selective PR modulators (SPRM) [18]. Docking and molecular similarity analysis studies have been performed on PR ligands belonging to quinoline derivatives [13], but no 3D pharmacophore hypothesis for hPR-A binding affinity has yet been explored. Subsequently, diverse sets of nonsteroidal derivatives are taken up to build robust QSAR model as well as to develop pharmacophore signal for selective PR-A binding affinity, which is further corroborated with interactions of the active ligand with the receptor at the binding pocket by structure-based drug design.

## Materials and Methods

In the present study, compounds (supplementary Table (Tab. S1)) containing nonsteroidal substituted quinoline [8–10, 19–22] and cyclocymopol monomethyl ether [7] derivatives expressing hPR-A binding affinity have been explored to generate QSAR model and design pharmacophore map using multiple linear regression [23] and receptor independent space modeling [24] techniques respectively. Binding affinity (K_i_, nM) [7–10, 19–22], expressed in terms of pK_i_ (log_10_10^4^/K_i_) has been considered as dependent variable for model generation. The common molecular scaffolds of (a) 1,2-dihydrochromeno[3,4-*f*] quinoline, (b and c) 6-aryl-1,2-dihydro-2,2,4-trimethylquinolines and (d) cyclocymopol monomethyl ether are depicted in Fig. 1. The models are validated by estimating *R^2^_pred_, R^2^_m_* and *se* (standard error of prediction) [25, 26] of test sets. Further the most active compound (cpd. **25** in supp. Tab. S1) of the data set is docked in protein crystal structure (pdb code: 2OVH) [27] to analyse the ligand-receptor interactions in 3-D space.

### QSAR study

Energy minimization of the 3D structure of compounds is performed in MOPAC module using the Austin Model 1 (AM1) to locate local minima conformers. The energy minimized structure is used to calculate different molecular properties, including physicochemical, electronic (atomic charge functions, orbital energies, partial charge function [28] using extended Hückel approach [29]), spatial, topological (E-state indices [30] and R-state indices [31]) properties, molecular geometries (geometrical, WHIM, 3D-MoRSE, molecular profiles, etc.) and structural features of the atoms [32]. The tools used to generate 3D structural descriptors are Chem3D Pro [29], CAChe [33], TSAR [34], ETSA-CA [35], MOE 2007.09 [36] and DRAGON 5.5 [37, 38]. The models are developed by standard and forward stepwise regression methods using Statistica 5.0 [39]. To obtain a robust and dependable model, the dataset of ligands is divided into modeling (n=130) and validation (Vs, *n*_Vs_=40) sets using sphere exclusion algorithm based on Euclidean distance [40]. Further the modeling set is splitted into training (Tr, *n*_Tr_=100) and test (Ts, *n*_Ts_=30) sets through the same principle, considering the most active and least active compounds present in training set. The following statistical parameters are used to evaluate the statistical significance of the regression equation: correlation coefficient (*R^2^*), standard error of estimate (*se*), explained variance (*EV*), variance ratio (*F*), degree of freedom (*df*) and average of absolute value of calculated residuals (*AVRES*). The predictive power of the model is estimated by cross-validated variance (*Q*^2^) (by leave one out method) [41], predictive residual sum of squares (*PRESS*), standard deviation error of prediction (*SDEP*) and average of absolute value of predicted residuals (*Pres_av_*). The model is further validated with test and validation sets, estimating *R^2^_pred_* and *R^2^_m_* [25, 26].

### Pharmacophore space modeling study

Receptor-independent space modeling study [24] generates pharmacophore hypothesis using Catalyst 4.11 [42] that can highlight on ligand-receptor interactions. For hypothesis generation, the dataset division is processed through the sphere exclusion approach [40], except input data for number of compounds of training set is fixed to ‘26’. The whole dataset is splitted into training (Tr, *n*_Tr_=26), test (Ts, *n*_Ts_=60) and validation (Vs, *n*_Vs_=84) sets and are fitted in the pharmacophore model and subsequently predicted the activity to adjudge the robustness of the hypothesis. The chemical features used for pharmacophore mapping are hydrogen bond (HB) acceptor (a) and donor (d), hydrophobic (p) and aromatic ring (r). Different control parameters employed for hypothesis generation (Hypogen process) are uncertainty, weight variation and spacing (minimum interfeature distance for hypothesis). Weight variation signifies the extent to which each feature contributes towards compound’s activity in the process of hypothesis generation, whereas uncertainty denotes the standard deviation of error cost, the deviation between the actual and the estimated activity of the compounds in training set. The overall cost of a hypothesis is obtained by summing up three cost factors: a weight cost, an error cost and a configuration cost. Weight cost is a value that increases as the weight variation of the model varies. The entropy of the hypothesis space is equal to the configuration cost, which is dependent on the complexity of the hypothesis space being optimized. The hypothesis estimates the costs of null and fixed hypothesis and the greater the difference, it is more likely that the hypothesis does not reflect a chance correlation. Lesser the value between fixed cost and total cost, better the hypothesis as it is more towards the ideal hypothesis. For the purpose of hypothesis optimization, the difference between total and null costs is considered to be 60 bits [43]. Two other factors considered for evaluating the pharma-cophore map are rmsd and correlation. Rmsd (root mean square deviation) indicates the quality of prediction for training set and correlation value derived from the geometric fit index. The generated hypothesis is further judged to nullify over-prediction of inactive ligands, using hyporefine process [42], where steric feature is also considered for bioactivity. The selected hypothesis is validated through a cross-validation technique using CatScramble based on Fischer’s randomization test [23] by random reassigning the activity values among the training set compounds. The predictive ability of the pharmacophore model is further screened with the estimated activity of test and validation sets compounds.

### Docking study

Receptor-based molecular docking study highlights the binding interaction at the active site residues [44]. Crystal structure of PR-A ligand binding domain (pdb code: 2OVH) [27] complexed with asoprisnil [45] and the corepressors SMRT has been obtained from RCSB protein data bank [46]. The docking study has been performed in Discovery Studio 1.7 [47] by using LigandFit of ‘Receptor-ligand interactions’ protocol. Pre-treatment process for both the active ligand (comp **25** in supp. Tab. S1) and the receptor are performed with ligand preparation and binding site definition. Constraint parameters used for ligand preparation are ionization change, tautomer and isomer generation; Lipinski filter and 3D generator albeit all the duplicate structures are removed. Receptor preparation is accomplished by defining the active site cavity with the aid of pre-existing ligand. The whole receptor is selected and hydrogen atoms are added to it. pH value of the protein has been set in the range of 6.5 to 8.5. The receptor-ligand interaction is explored with LigandFit optimization utilizing dreiding as the energy grid force-field; Monte Carlo trial method for conformational search with consideration of electrostatic energy, torsional step size for polar hydrogen at 30, maximum internal energy at 10^4^ kcal/mol and maximum poses of 10 in docking mode [48]. During docking of the ligands, geometry optimization of the receptor-ligand complex is not performed due to preserve native form of the ligand-bound receptor. The scoring parameters (LigScore, PLP, Jain, PMF and Ludi energy estimate) are used for analysis. Finally the docked receptor-ligand complex is analyzed to investigate the type of interactions and compare dock score.

### Virtual screening and molecular docking studies

*In silico* screening is a rapid technique to obtain hit compounds with desired activity profiles [49]. The validated pharmacophore model has been used to screen WDI (World Drug Index) (NCI, Maybridge, ZINC) libraries comprising ∼ 10,000,000 compounds in order to calculate the rate of recovering the experimental hits from the primary screening library. Fast flexible search algorithm is used for database screening. Out of 10,000 compounds retrieved from each database, the hits are narrowed down to <100 compounds based on estimated activity (a cut off value of 0.32, K_i_ of comp **25**). Simultaneously the virtual screening is conducted using validated QSAR models, and hits are identified by consensus agreement between these models. The predictions are categorized by model coverage using Z cut-off of 0.2. Consensus molecular descriptors, used for model generation, are generated for the set of compounds and consensus activities are predicted from the proposed QSAR models. In order to access the ability of models to recover the active compounds from the screening library, three criteria, i.e. hit rate, yield and the enrichment factor are used [25]. Moreover, Lipinski’s rule of five is used to eliminate non-drug like compounds. Finally compounds of promising K_i_ are docked individually into the active site cavity of the receptor. The receptor-ligand complexes are investigated to find out important interactions at the receptor cavity as well as dock scores.

## Results and Discussion

### QSAR study

Different molecular properties, including physicochemical, topological, electronic, spatial descriptors are used for model generation. The best models obtained in different permutation of descriptors are given in Tab. 1.

**Fig. 2:**
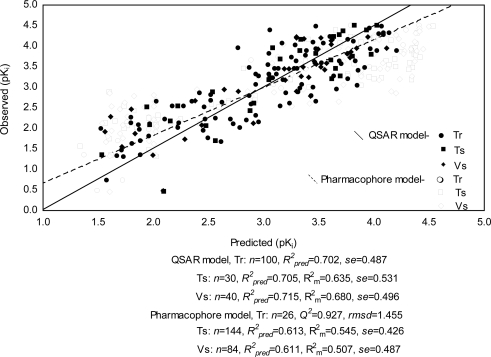
Observed vs predicted binding affinity of QSAR consensus and pharmacophore models.

All the QSAR models can explain for more than 72% variance in activity and cross-validated variance of 70%. The models have also good predictive property (R^2^_pred_ and R^2^_m_ > 0.50), except model I in validation set. All of the generated models are statistically significant and are analyzed for consensus prediction of Tr (*n*_Tr_=100, *R^2^_pred_*=0.702, *se*=0.487), Ts (*n*_Ts_=30, *R^2^_pred_*=0.705, *se*=0.531, *R^2^_m_* =0.635) and Vs (*n*_Vs_=40, *R^2^_pred_*=0.715, *se*=0.496, *R^2^_m_*=0.680), suggesting the robustness of models. The observed vs consensus predicted binding affinity of the compounds as per QSAR models is plotted in Fig. 2 and listed in supplementary Tab. S2. The acronyms used in the above models (I-V) for the descriptors are provided in Tab. 2.

Among the descriptors, IC3 and CIC3 depict the topological features of atoms based on neighborhood environment [38]. F07[C-O] and other 2D frequency fingerprint descriptors also describe topological features of molecules. 3-D arrangement of atoms, bond distances, ring types, planar, non-planar systems and atom types along with atomic polarizabilities are encoded by RDF130p and RDF125p [38]. The values of polarizability are dependent on the chemical environment of atoms and have great influence on bonds [50]. GATS8e [38] is a distance-type function that also includes atomic properties, e.g. electronegativity. It accounts for the correlation among atoms, weighted by atomic Sanderson electronegativity with a distance of eight bonds (the lag) in the molecule [51]. nOHs provides local chemical information that is insensitive to isomers and to conformational changes, and shows a high level of degeneracy [38, 52]. Mor03m, derived from infrared spectra simulation, suggests the relevance of atomic masses and the 3D atomic coordinates [38, 53]. The role of atomic information, Van der waals volumes relevant to the strength of ligand-receptor interaction and the molecular topology to the activity are described by BELv2 [38, 54]. As an inference from these complex descriptors, it can be suggested that presence of functional atoms, F, Cl, N and O have influence on ligand binding affinity depending on topology of the ligands, atomic polarizabilities and electronegativities, atomic masses and van der Waals volume.

### Pharmacophore space modeling study

Receptor independent pharmacophore mapping of the ligands is explored through standardization of the training set (Tr, *n*_Tr_=26) and its subsequent optimization utilizing the control parameters. The results of the optimization study based on the cost difference (Δcost), root mean square deviation (rmsd) and best correlation (Q^2^) are listed in Tab. 3.

**Fig. 3:**
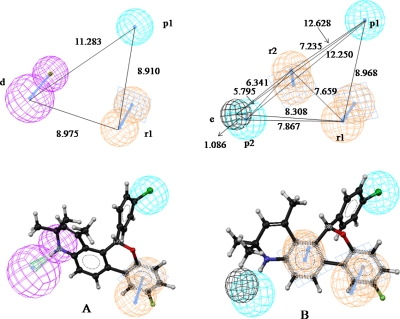
Pharmacophore features of run nos. 7 and 8 fitted with active ligand. (A) Hypogen hypothesis: *Q^2^*=0.909, *rmsd*=1.615, Δcost=146.075; (B) Hyporefine hypothesis: *R*=0.927, *rmsd*=1.455, Δcost=152.462; Features include hydrogen bond donor (d), hydrophobic (p), Ring aromatic (r) and excluded volume (e).

The optimized hypothesis (run no. 7) showed more than 90% correlation to binding affinity, whereas the hyporefine (run no. 8) of the same is observed to correlate 92.7% with activity with highest cost differences (Δcost) of 152.462 bits and low rmsd value of 1.455. The fixed and null costs are 90.748 and 270.771 bits respectively for both run nos. 7 and 8, but the difference between fixed and total costs is lower (27.561 bits) in run no. 8. Further the prediction sets for the best hypothesis (run no. 8), with *n*_Ts_=60, *R^2^_pred_*=0.613, *se*=0.426, *R^2^_m_*=0.545 and *n*_Vs_=84, *R^2^_pred_*=0.611, *se*=0.487, *R^2^_m_*=0.507, have been found to be superior than run no. 7. The observed and predicted binding affinities (pK_i_) of the compounds are represented in Fig. 2 and tabulated in supplementary Tab. S2. The quality of hypotheses generated for binding affinity are adjudged by a cross-validation technique using Fischer’s randomization test [23] at the 99% confidence level, but no hypothesis generated better parameters than the original hypothesis of run nos. 7 and 8 in either case. Both the hypotheses (run nos.7 and 8) are taken into consideration for describing the pharmacophore features of the dataset and docking interactions with the most active ligand (comp **25** in supp. Tab. S1), and observed that presence of HB donor (d), hydrophobic (p1) and aromatic ring (r1) features in run no. 7 and two hydrophobic (p1 and p2), two aromatic ring (r1 and r2) features along with steric influence (e) in run no. 8 are essential for effective binding. The mapped pharmacophore features and inter-feature distances (Å) of the both hypotheses for binding affinity to hPR-A are depicted in Fig. 3. Both the hypotheses illustrate presence of electronegative substitution at para position of 5-aryl ring (Fig. 1a) offers hydrophobic region. SAR study on chromeno quinolines (Fig. 1a) also adjudged the para substitution in 5-aryl moiety is one of the essential biophore for hPR agonist activity [9]. Presence of N-hetero atom in ring A (Fig. 1a–1c) as HB donor has been found to be a significant contributor in binding affinity, which is due to overlapping similarity of the A-ring of quinoline with steroid D-ring [9]. However HB donor is reverted to steric and hydrophobic regions due to unavailability of N-atom in the ring (Fig. 1d), when weightage is given to excluded volume (hyporefine, run no. 8). Additionally mapping also demonstrates presence of aromatic rings B and D in scaffold (Fig. 1a–1c) have positive impact on bioactivity. Halogen substituents in D-ring are demonstrated to be critical pharmacophore feature for receptor binding, adjudged by the SAR study [8]. Pharmacophore mapping outcomes can be corroborated with the QSAR study, which also thrust on presence of nitrogen hetero atom in ring A, influence of para-substituted 5-aryl moiety as well as the rings B and D for bioactivity. The reliability of the receptor-independent pharmacophore map is validated in light of binding site interactions of the most active ligand at the active site cavity of the receptor.

### Binding interactions at the active site

**Fig. 4:**
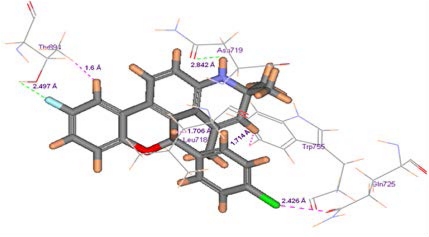
Active ligand at the binding site of 2OVH [27]. Catalytic residues are labeled.

The most active ligand (comp **25** in supp. Tab. S1) of the molecular dataset is considered for docking in the catalytic cleft of the receptor (2OVH) [27] in order to explore the binding modes in relation to mapped biophore. The binding interactions of the compound are portrayed in Fig. 4. The amino acids responsible for vital interactions with the ligand are Asn719, Thr894 (polar amino acids) and Leu718, Gln725, Trp755 (non-polar amino acids). Nitrogen hetero atom present in the ring A (Fig. 1a) of the docked ligand forms HB interaction with amino acid Asn719 in the catalytic cleft at a distance of 2.842 Å, whereas the para-halo substituent of 5-aryl ring forms van der Waals interaction with the polar catalytic residue Gln725 at a distance of 2.426 Å. Electronegative substituent in D ring also forms HB interaction with Thr894 at distance of 2.497 Å, and steric association of D-ring is seen at 1.6 Å with the same amino acid residue in the binding pocket respectively. It is also observed that Leu718 and Trp755 interact with the hydrophobic aromatic core of rings A and C at 1.706 and 1.714 Å distance respectively with steric bumps. The PMF score (Potential of Mean Force) [55] of the docked ligand has been found to be −89.12. It is revealed from the analysis that the most active compound has comparable dock score as that of the standard ligands (progesterone −80.329, medroxy progesterone acetate −94.52, mifepristone −83.045 and norethindrone −65.564) and explains for good binding affinity of the ligand in active site of receptor. The binding interactions are further adjudged with the pharmacophore model that indicates the functional atoms, nitrogen in ring A of quinoline scaffold (Fig. 1a–1c) behaves as promising HB donor, and chlorine in 5-aryl ring as hydrophobic zone, have binding interactions with catalytic residues, Asn719 by hydrogen bonds and Gln725 by hydrophobic interaction respectively. Aromatic feature of ring D of quinoline offers core for hydrophobic interactions with Thr894 at the active site cavity. Hydrophobic interactions are also observed between rings A and C with Trp755 and Leu718. The docking study of the ligand adjudges the pharmacophore map fairly. These findings are compliant with SPRM binding analysis that revealed the importance of electrostatic, HB donor, and hydrophobic properties of ligand for interaction with catalytic residues, Leu718, Asn719 and Gln725 [13].

### Virtual screening and molecular docking studies

The hit compounds which satisfied the screening criteria using validated QSAR and pharmacophore models are reported in Tab. 4. Consensus predicted activity from QSAR and pharmacophore models of NCI0101316 are found to be 0.12 and 0.22 nM respectively. The mean PMF dock score is −86.41.Second compound NCI0023681 depicts predicted activity of 0.38 and 0.18 from QSAR consensus and pharmacophore models, along with PMF dock score of −70.31. NCI0050131 provides consensus predicted activity of 0.87 and estimated activity of 0.22 from pharmacophore model and the docking score is −78.86. The results are further adjudged with binding interactions with the catalytic residues in the receptor cavity.

Docking studies reveal crucial binding interactions at the active site cavity of the receptor. NCI0101316 interacts with Asn719, Cys891 (polar amino acids) and Met756 (non-polar amino acids), forming HB and hydrophobic interactions with Asn719 at 1.767 and 1.667 Å respectively, whereas catalytic amino acid residues Cys891 and Met756 interacts at 1.922 and 1.831 Å distances respectively with steric bumps. NCI0023681 forms HB interactions with Asn719, Leu887 and Thr894 at distances of 1.780, 2.377 and 2.299 Å respectively and hydrophobic associations with Leu718, Asn719 and Met756 at distances of 1.796, 1.415 and 1.788 Å respectively. NCI0050131 binds to catalytic residues Leu718 and Thr894 through van der Waals interaction and HB interaction at 1.912 and 2.202 Å respectively (supplementary figure Fig. F1). These interactions are found to be vital with respect to asoprisnil (pre-existing ligand of the receptor) as well as SPRM binding studies, consequently these three compounds are proposed to be showing good binding affinity to hPR as per the models suggested.

## Conclusion

Ligand-based molecular modeling studies are investigated on nonsteroidal quinoline and cyclocymopol monomethyl ether derivatives to generate models for exploring unique pharmacophore features of SPRMs. QSAR and pharmacophore space modeling studies developed statistically significant models and validated internally and externally with test set compounds. The space modeling map is in conformity with the molecular descriptors giving insight on the importance of functional atoms (O, N, Cl, F), polarizability, van der waals volume for presence of HB donor, hydrophobic, steric and aromatic ring attributes for ligand fitting in the active site cavity of the receptor with catalytic receptor residues. Finally three compounds are proposed to be newer analogs with significant binding affinity to PR-A as per the molecular modeling studies.

## Supporting Information



## Figures and Tables

**Tab. 1.** Statistical quality of best QSAR models.

**Model no.**	**Descriptors**	**Training set (*n*_Tr_=100)**	**Test set (n_Ts_=30)**	**Validation set (*n*_Vs_=40)**
I	IC3, F07[C-O], RDF130p, F10[F-F], F04[F-F], GATS8e	*R^2^*=0.774, *EV*=75.9%, *se*=0.451, *F*=53.05, *df*=6, 93, *AVRES*=0.795, *PRESS*=21.799, *SDEP*=0.467, *Pres_av_*=0.705, *Q^2^*=0.740	*R^2^_pred_*=0.696 *se*=0.565 *R^2^_m_*=0.708	*R^2^_pred_*=0.458 *se*=0.660 *R^2^_m_*=0.422
II	F04[C-C], F05[C-Cl], RDF130p, F07[C-O], GATS8e, F10[F-F]	*R^2^*=0.737, *EV*=72%, *se*=0.474, *F*=43.35, *df*=6, 93, *AVRES*=0.765, *PRESS*=23.881, *SDEP*=0.489, *Pres_av_*=0.630, *Q^2^*=0.699	*R^2^_pred_*=0.694 *se*=0.569 *R^2^_m_*=0.692	*R^2^_pred_*=0.672 *se*=0.546 *R^2^_m_*=0.668
III	F04[C-C], CIC3, nOHs, Mor03m, RDF125p	*R^2^*=0.759, *EV*=74.6%, *se*=0.448, *F*=59.08, *df*=5, 94, *AVRES*=0.757, *PRESS*=21.193, *SDEP*=0.460, *Pres_av_*=0.643, *Q^2^*=0.729	*R^2^_pred_*=0.656 *se*=0.576 *R^2^_m_*=0.604	*R^2^_pred_*=0.642 *se*=0.565 *R^2^_m_*=0.645
IV	F04[C-C], CIC3, nOHs, Mor03m, BELv2, RDF125p	*R^2^*=0.775, *EV*=76.1%, *se*=0.440, *F*=53.53, *df*=6, 93, *AVRES*=0.769, *PRESS*=20.551, *SDEP*=0.453, *Pres_av_*=0.656, *Q^2^*=0.744	*R^2^_pred_*=0.621 *se*=0.605 *R^2^_m_*=0.545	*R^2^_pred_*=0.676 *se*=0.539 *R^2^_m_*=0.677
V	F04[C-C], CIC3, nOHs, Mor03m, F04[N-N], RDF125p	*R^2^*=0.773, *EV*=75.9%, *se*=0.441, *F*=52.91, *df*=6, 93, *AVRES*=0.765, *PRESS*=20.857, *SDEP*=0.457, *Pres_av_*=0.658, *Q^2^*=0.738	*R^2^_pred_*=0.658 *se*=0.577 *R^2^_m_*=0.599	*R^2^_pred_*=0.662 *se*=0.547 *R^2^_m_*=0.665

**Tab. 2.** Symbols and function of the descriptors.

**Symbols**	**Types of descriptors**	**Description**
IC3	Information indices	information content index (neighborhood symmetry of 3-order)
F07[C-O]	2D frequency fingerprints	frequency of C-O at topological distance 7
RDF130p	RDF descriptors	Radial Distribution Function −13.0 / weighted by atomic polarizabilities
F10[F-F]	2D frequency fingerprints	frequency of F-F at topological distance 10
F04[F-F]	2D frequency fingerprints	frequency of F-F at topological distance 4
GATS8e	List of 2D autocorrelation indices	Geary autocorrelation -lag 8 / weighted by atomic Sanderson electronegativities
F04[C-C]	2D frequency fingerprints	frequency of C-C at topological distance 4
F05[C-Cl]	2D frequency fingerprints	frequency of C-Cl at topological distance 5
CIC3	Information indices	complementary information content (neighborhood symmetry of 3-order)
nOHs	Functional group counts 3D-MoRSE (3D Molecule	number of secondary alcohols
Mor03m	Representation of Structures based on Electron diffraction) descriptors	3D-MoRSE - signal 03 / weighted by atomic masses
RDF125p	RDF descriptors	Radial Distribution Function −12.5 / weighted by atomic polarizabilities
BELv2	Burden eigenvalue descriptors	lowest eigenvalue n. 2 of Burden matrix / weighted by atomic van der Waals volumes
F04[N-N]	2D frequency fingerprints	frequency of N-N at topological distance 4

**Tab. 3.** Hypothesis parameters observed in pharmacophore study.

**Run no.**	**UC**	**WV**	**Spacing (pm)**	**Pharmacoph. features in generated hypothesis**	**Cost**					**Q^2^**	**rmsd**
					**Null**	**Fixed**	**Total**	**Δcost**	**Config**		
1	3	0.302	300	p_1_, p_2_, r_1_	165.194	101.666	117.38	47.814	13.0868	0.905	1.047
2	3	0.302	250	p_1_, p_2_, r_1_, r_2_	165.194	102.212	114.696	50.498	13.634	0.919	0.964
3	3	1.5	300	p_1_, p_2_, r_1_	165.194	102.467	115.78	49.414	13.087	0.912	1.005
4	3	2.5	300	p_1_, p_2_, r_1_	165.194	102.722	116.357	48.837	13.087	0.908	1.022
5	2.5	0.302	300	p_1_, p_2_, r_1_	194.491	96.947	118.905	75.586	13.087	0.906	1.246
6	2	0.302	300	p_1_, r_1_, r_2_	270.771	89.691	128.379	142.392	13.087	0.899	1.698
7	2	2.5	300	d_1_, p_1_, r_1_	270.771	90.748	124.696	146.075	13.087	0.909	1.615
8^*^	2	2.5	300	p_1_, p_2_, r_1_, r_2_, e	270.771	90.748	118.309	152.462	13.087	0.927	1.455

Input features: Hydrogen bond donor (d), Hydrophobic (p), Ring aromatic (r), excluded volume (e); Δcost = Null cost – Total cost, UC = uncertainty, WV = weight variation, Config. = configuration cost, Q^2^=cross-validated variance, rmsd= root mean square deviation;

* Hyporefine of run no. 7.

**Tab. 4.** Proposed compounds obtained from virtual screening and docking studies.

**Compound name**	**SMILES**	**Consensus predicted activity (QSAR model) (K_i_, nM)**	**Estimated activity (Pharma-cophore model) (K_i_, nM)**	**Dock score (PMF)**	**Binding interactions**	
					**HB interaction**	**Hydrophobic interaction**
NCI0101316	O=C4N([H])C(=O)[C@]5([H])[C@]1([H])C(=C([H])[C@@]([H])(C1=C(C=2/N=C(/[H])C([H])=C([H])C=2[H])C3=C([H])C([H])=C([H])C([H])=C3[H])[C@]45[H])[C@@](O[H])(C6=C([H])C([H])=C([H])C([H])=C6[H])C=7/N=C(/[H])C([H])=C([H])C=7[H]	0.12	0.22	–86.41	Asn719	Asn719, Cys891 and Met756
NCI0023681	[H]C([H])(N([H])C([H])([H])C1=C([H])C([H])=C([H])C([H])=C1[H])[C@]2([H])[C@@]([H])(O[H])C([H])([H])C([H])([H])[C@@]6([H])[C@]2([H])C([H])([H])[C@@]5([H])C4=C(/C3=C(\[H])C([H])=C([H])C([H])=C3N4[H])C([H])([H])C([H])([H])N5C6([H])[H]	0.38	0.18	–70.31	Asn719, Leu887 and Thr894	Leu718, Asn719 and Met756
NCI0050131	O=C(O[H])[C@]2([H])[C@]([H])(C1=C(C([H])=C(OC([H])([H])[H])C(OC([H])([H])[H])=C1[H])[C@@]([H])(O[H])[C@]2(O[H])C([H])([H])O[H])C=3C([H])=C(OC([H])([H])[H])C(OC([H])([H])[H])=C(OC([H])([H])[H])C=3[H]	0.87	0.22	–78.86	Thr894	Leu718

## Supporting Information

Structural features and binding affinity (K_i_) of hPR-A modulators are described in Tab. S1. Observed and predicted activities (pK_i_) of compounds are listed in Tab. S2. The binding interactions at the catalytic cleft with hit ligands are portrayed in Fig. S1. These documents are available in the online version (Format: PDF, Size: < 0.3 MB): http://dx.doi.org/10.3797/scipharm.1105-03
