# Supplementary material for: Molecular Modeling on Structure-Function Analysis of Human Progesterone Receptor Modulators
Source: Sci Pharm. 2011 Jun 30;79(3):461–77. doi: 10.3797/scipharm.1105-03 (PMC3163366; doi:10.3797/scipharm.1105-03)
Supplement: Supplementary file 1 [file Scipharm-2011-79-461supportinginformation.pdf]

Scientia Pharmaceutica www.scipharm.at

Open Access

Supporting Information to

Molecular Modeling on Structure-Function Analysis of Human Progesterone Receptor Modulators

Ria PAL, Md Ataul ISLAM, Tabassum HOSSAIN, Achintya SAHA

Published in Sci Pharm. 2011; 79: 461–477 doi:10.3797/scipharm.1105-03

Available from: http://dx.doi.org/10.3797/scipharm.1105-03

© Pal *et al.*; licensee Österreichische Apotheker-Verlagsgesellschaft m. b. H., Vienna, Austria.

This is an Open Access article distributed under the terms of the Creative Commons Attribution License (http://creativecommons.org/licenses/by/3.0/), which permits unrestricted use, distribution, and reproduction in any medium, provided the original work is properly cited.

Table of Contents

Tab. S1. Structural features and binding affinity (K_i) of hPR-A modulators

Tab. S2. Observed and predicted activities (pK_i) of compounds

Fig. S1. Hit ligands at the binding site of 2OVH [64]. Catalytic residues are labeled.

Tab. S1. Structural features and binding affinity (K_i) of hPR-A modulators

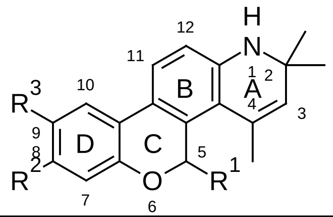

Cpd no.	R1	R2	R3	R4	R5	R6	R7	R8	K_i (nM)
1	H	H	H	–	–	–	–	–	84.00
2	CH_3	H	H	–	–	–	–	–	3.30
3	$(CH_2)_3CH_3$	H	H	–	–	–	–	–	0.95
4	$(CH_2)_5CH_3$	H	H	–	–	–	–	–	23.00
5	$(CH_2)_4Cl$	H	H	–	–	–	–	–	3.20
6	$CH_2CH{=}CH_2$	H	H	–	–	–	–	–	4.80
7	H	H	F	–	–	–	–	–	6.10
8	CH_3	H	F	–	–	–	–	–	1.10
9	$(CH_2)_3CH_3$	H	F	–	–	–	–	–	2.90
10	H	H	Cl	–	–	–	–	–	3.60
11	$(CH_2)_3CH_3$	H	Cl	–	–	–	–	–	0.87
12	H	H	CH_3	–	–	–	–	–	50.20
13	$(CH_2)_3CH_3$	H	CH_3	–	–	–	–	–	1.20
14	$(CH_2)_3CH_3$	H	OCH_3	–	–	–	–	–	3.50
15	$(CH_2)_3CH_3$	F	H	–	–	–	–	–	26.50
16	$O(CH_2)_2CH_3$	H	H	–	–	–	–	–	64.00
17	OCH_3	H	F	–	–	–	–	–	38.40
18	$O(CH_2)_2CH_3$	H	F	–	–	–	–	–	6.00
19	OCH_3	H	Cl	–	–	–	–	–	16.40
20	$O(CH_2)_2CH_3$	H	Cl	–	–	–	–	–	1.50
21	OCH_3	F	H	–	–	–	–	–	80.50
22	$S(CH_2)_2CH_3$	H	H	–	–	–	–	–	24.30
23	$S(CH_2)_2CH_3$	H	F	–	–	–	–	–	6.60

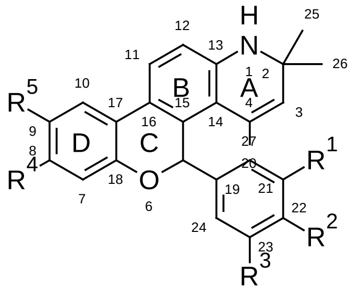

Cpd no.	R1	R2	R3	R4	R5	R6	R7	R8	K_i (nM)
24	H	Cl	H	H	H	–	–	–	0.70
25	H	Cl	H	H	F	–	–	–	0.32
26	H	Cl	H	F	H	–	–	–	2.70
27	H	Cl	H	H	Cl	–	–	–	0.59
28	H	Cl	H	H	OCH_3	–	–	–	2.40
29	H	H	H	H	F	–	–	–	2.20
30	H	H	Cl	H	F	–	–	–	0.32

Tab. S1. (Cont.)

Cpd no.	R^1	R^2	R^3	R^4	R^5	R^6	R^7	R^8	K_i (nM)
31	H	Cl	CH_3	H	F	–	–	–	0.49
32	H	OCH_3	H	H	F	–	–	–	2.70
33	H	H	CH_3	H	F	–	–	–	0.37
34	H	H	CF_3	H	F	–	–	–	0.78
35	H	F	CH_3	H	F	–	–	–	1.30
36	H	H	H	H	Cl	–	–	–	0.74
37	H	Br	H	H	Cl	–	–	–	0.59
38	H	OCH_3	H	H	Cl	–	–	–	0.93
39	H	H	Cl	H	Cl	–	–	–	0.48
40	H	H	CH_3	H	Cl	–	–	–	0.55
41	H	H	CF_3	H	Cl	–	–	–	1.10
42	H	H	F	H	Cl	–	–	–	0.34
43	H	Cl	CH_3	H	Cl	–	–	–	0.50
44	H	F	CH_3	H	Cl	–	–	–	1.30
45	H	H	H	H	H	–	–	–	3.60
46	H	F	H	H	H	–	–	–	5.30
47	H	Br	H	H	H	–	–	–	0.55
48	H	H	F	H	H	–	–	–	0.69
49	H	H	Cl	H	H	–	–	–	0.43
50	H	H	Br	H	H	–	–	–	1.10
51	H	CF_3	H	H	H	–	–	–	10.80
52	H	$COCH_3$	H	H	H	–	–	–	8.80
53	H	CH_3	H	H	H	–	–	–	2.30
54	H	OCH_3	H	H	H	–	–	–	13.30
55	H	H	CF_3	H	H	–	–	–	2.60
56	H	Cl	Cl	H	H	–	–	–	3.40
57	H	Cl	F	H	H	–	–	–	0.41
58	H	Cl	CH_3	H	H	–	–	–	1.20
59	H	F	CF_3	H	H	–	–	–	6.80
60	H	CH_3	F	H	H	–	–	–	1.80
61	Cl	H	Cl	H	H	–	–	–	1.70
62	F	H	Br	H	H	–	–	–	2.40
63	CH_3	H	Br	H	H	–	–	–	0.87
64[a]	H	Br	H	H	H	–	–	–	3.90
65[b]	H	H	Br	H	H	–	–	–	13.80
66[c,d]	H	Cl	H	H	H	–	–	–	11.60
67[d]	H	Cl	H	H	H	–	–	–	10.10
68[d,e]	H	Cl	H	H	H	–	–	–	24.60
69[f]	H	H	H	H	H	–	–	–	80.00
70[d,g]	H	Cl	H	H	H	–	–	–	1.30
71[d,g]	H	H	H	H	H	–	–	–	28.60
72[d,g]	H	H	H	H	H	–	–	–	6.60
73[d,g]	H	H	F	H	H	–	–	–	2.60
74[d,g]	H	H	CF_3	H	H	–	–	–	16.60
75[d,g,h]	H	Cl	H	H	H	–	–	–	5.30

Tab. S1. (Cont.)

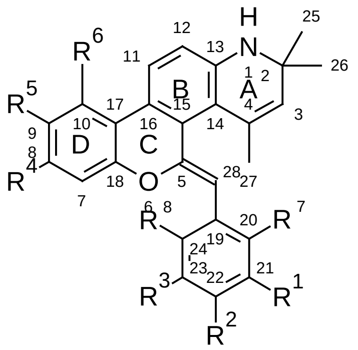

Cpd no.	R^1	R^2	R^3	R^4	R^5	R^6	R^7	R^8	K_i (nM)
76	H	H	H	H	H	H	CH_3	H	0.66
77	H	H	H	H	H	F	H	H	0.61
78	F	H	H	H	H	F	H	H	5.50
79	H	F	H	H	H	F	H	H	0.71
80	H	H	H	H	H	F	CH_3	H	1.50
81	H	H	H	F	H	H	H	H	0.62
82	H	H	H	F	H	H	CH_3	H	0.55
83	H	H	H	F	H	H	CH_2CH_3	H	2.30
84	H	H	H	F	H	H	$CH_2CH_2CH_3$	H	1.60
85	H	H	H	F	H	H	$CH(CH_3)_2$	H	1.90
86	H	H	H	F	H	H	OCH_3	H	3.80
87	H	H	H	F	H	H	SCH_3	H	0.59
88	H	H	H	F	H	H	F	H	0.62
89	H	H	H	F	H	H	Cl	H	22.40
90	H	H	H	F	H	H	Br	H	16.40
91	H	H	H	F	H	H	CHO	H	2.60
92	H	H	H	F	H	H	OCF_3	H	6.40
93[i]	H	H	H	F	H	H	H	H	1.50
94[i]	H	H	H	F	H	H	CH_3	H	1.40
95[j]	H	H	H	F	H	H	H	H	9.20
96[j]	H	H	H	F	H	H	H	CH_3	0.42
97	H	H	H	F	H	H	$N(CH_3)_2$	H	1.20
98	F	H	H	H	H	H	H	H	0.83
99[d]	F	H	H	H	H	H	H	H	6.30
100[d,g]	F	H	H	H	H	H	H	H	3.50
101[d,g]	H	H	H	H	H	H	H	H	4.90
102[k]	H	H	H	H	H	H	H	H	2.10
103	H	H	H	H	H	H	H	H	4.90
104	H	H	H	H	H	H	F	H	5.60
105	H	F	H	H	H	H	H	H	3.50
106	H	H	H	H	H	H	Br	H	5.50
107	H	Br	H	H	H	H	H	H	8.70
108	H	H	H	H	H	H	Cl	H	1.30
109	H	Cl	H	H	H	H	H	H	60.00
110	H	F	H	H	H	H	F	H	3.20

Tab. S1. (Cont.)

Cpd no.	R^1	R^2	R^3	R^4	R^5	R^6	R^7	R^8	K_i (nM)
111	H	H	F	H	H	H	F	H	1.50
112	H	H	H	H	H	H	F	F	31.30
113	F	F	H	H	H	H	H	H	3.60
114	F	H	F	H	H	H	H	H	12.60

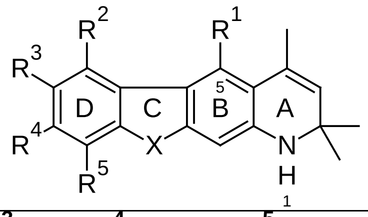

Cpd no.	R^1	R^2	R^3	R^4	R^5	X	K_i (nM)
115	H	H	H	H	H	–	133.00
116	H	H	H	H	H	CH_2	14.00
117	H	H	H	H	CH_2OH	CH_2	12.40
118	H	H	H	$COCH_3$	H	CH_2	176.00
119	H	H	H	Br	H	CH_2	24.00
120	H	H	H	Cl	H	CH_2	29.00
121	H	F	H	H	H	CH_2	11.20
122	H	H	H	H	H	O	184.00
123	H	H	H	H	H	NH	113.00
124	H	H	H	H	H	C=O	3553.00
125	H	F	H	H	H	C=O	29.10
126[l]	H	H	H	H	H	CH_2	87.00
127	H	H	H	H	H	C(H)OH	483.00
128	H	H	H	Br	H	C(H)OH	1858.00
129	H	H	H	F	H	C(H)OH	101.00
130	H	F	H	H	H	C(H)OH	449.00
131	Cl	H	H	H	H	C(H)OH	53.10
132	H	H	F	H	F	C(H)OH	202.00
133	F	H	H	F	H	$C(H)OCOCF_3$	62.50

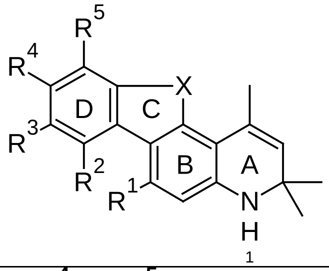

Cpd no.	R^1	R^2	R^3	R^4	R^5	X	K_i (nM)
134	H	H	H	H	H	CH_2	13.00
135	H	H	H	NO_2	H	CH_2	57.00
136	H	H	H	Br	H	CH_2	200.00
137	H	H	H	H	F	CH_2	15.50

Tab. S1. (Cont.)

Cpd no.	R^1	R^2	R^3	R^4	R^5	X	K_i (nM)
138	H	H	F	NO_2	H	CH_2	97.00
139	F	H	H	F	H	CH_2	20.90
140	H	H	H	H	H	O	77.00
141	H	H	H	H	H	$NCH_2CH_2CH_2CH_3$	77.00

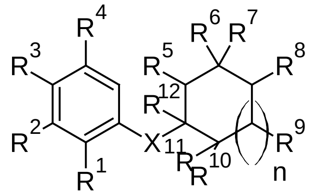

Cpd.	R^1	R^2	R^3	R^4	R^5	R^6	R^7	R^8	R^9	R^{10}	R^{11}	$R^1{}_2$	X	n	K_i (nM)
142	OH	H	Br	OCH_3	$=CH_2$	H	H	H	$-Br^m$	$-CH_3^n$	$-CH_3^m$	H	$CH_2(R)$	1	490.00
143	OH	H	Br	OCH_3	$=CH_2$	H	H	H	$-Br^m$	$-CH_3^n$	$-CH_3^m$	H	$CH_2(S)$	1	343.00
144	OH	H	Br	OCH_3	$=CH_2$	H^o		H	H	$-CH_3^n$	$-CH_3^m$	H	CH_2	1	77.00
145	OH	H	Br	OCH_3	$=CH_2$	H^p		H	H	$-CH_3^n$	$-CH_3^m$	H	CH_2	1	156.00
146	OH	H	Br	OCH_3	$=CH_2$	H^o		CH_3	H	$-CH_3^n$	$-CH_3^m$	H	CH_2	1	31.00
147	OH	H	Br	OCH_3	$=CH_2$	H^o		H	-	$-CH_3^n$	$-CH_3^m$	H	CH_2	0	441.00
148	OH	H	Br	OCH_3	$=CH_2$	H	H	$-CH_3^n$	H	$-CH_3^n$	$-CH_3^m$	H	$-CH_2^n$	1	22.00
149	OH	H	Br	OCH_3	$=CH_2$	H	H	$-CH_2OH^n$	H	$-CH_3^n$	$-CH_3^m$	H	$-CH_2^n$	1	3247.00
150	$OCOCH_3$	H	Br	OCH_3	$=CH_2$	H^p		CH_3	H	$-CH_3^n$	$-CH_3^m$	H	CH_2	1	59.60
151	$OCOCH_3$	H	Br	OCH_3	Δ	H^p		CH_3	H	$-CH_3^n$	$-CH_3^m$	H	CH_2	1	98.00
152	OH	H	Br	OCH_3	$CH_3{}^p$	H		$=CH_2$	H	$-CH_3^n$	$-CH_3^m$	H	CH_2	1	109.00
153	OH	H	Br	OCH_3	$=CH_2$	H^p		CH_3	H	$-CH_3^n$	$-CH_3^m$	H	$-CH=CH-CH_2$	1	84.00
154	H	H	NO_2	CH_3	$=CH_2$	H^p		CH_3	H	$-CH_3^n$	$-CH_3^m$	H	CH_2	1	28.80
155	H	H	NO_2	H	$=CH_2$	H^p		CH_3	H	$-CH_3^n$	$-CH_3^m$	H	CH_2	1	53.50
156	H	H	NO_2	H	$=CH_2$	H^p		CH_3	H	$-CH_3^n$	$-CH_3^m$	H	S	1	119.00
157	H	H	NO_2	H	$=CH_2$	H^p		CH_3	H	$-CH_3^n$	$-CH_3^m$	OH	CH_2	1	243.00
158	H	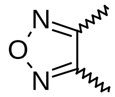		H	$=CH_2$	H^p		CH_3	H	$-CH_3^n$	$-CH_3^m$	H	CH_2	1	88.00
159	OH	H	Br	OCH_3	$=CH_2$	H	H	H	H	$-CH_3^n$	$-CH_3^m$	H	$CH_2(R)$	1	218.00
160	OH	H	Br	OCH_3	$=CH_2$	H	H	H	H	$-CH_3^n$	$-CH_3^m$	H	$CH_2(S)$	1	34.00
161	OH	H	Br	OCH_3	$=CH_2$	$-CH_3^n$	$-OH^n$	H	H	$-CH_3^n$	$-CH_3^m$	H	$-CH_2^n$	1	275.00
162	$OCOCH_3$	H	Br	OCH_3	$=CH_2$	CH_3^p		H	H	$-CH_3^n$	$-CH_3^m$	H	$-CH_2^n$	1	455.00
163	OH	H	Br	OCH_3	$=CH_2$	H	H	H	H	$=CH_3^q$		H	$CH_2(R)$	1	71.00
164	OH	H	Br	OCH_3	$=CH_2$	H	H	H	H	$=CH_3^q$		H	$CH_2(R)$	1	69.00
165	$OCOCH_3$	H	Br	OCH_3	$=CH_2$	H^p		CH_3	H	$-CH_3^m$		H	$-CH_2^n$	1	21.00
166	OH	H	Br	OCH_3	$=CH_2$	H^p		CH_3	H	$-CH_3^m$		H	$-CH_2^n$	1	84.00

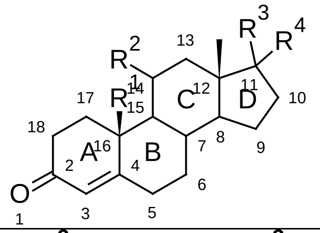

Cpd. No.	R^1	R^2	R^3	R^4	K_i (nM)
167	$-CH_3$	-H	-H	$-COCH_3$	3.50
168	$-CH_3$	-H	$-OCOCH_3$	$-COCH_3$	0.34
169	-H	$-C_6H_5N(CH_3)_2$	-OH	$-CCCH_3$	1.10
170	-H	-H	-OH	-CCH	1.87

Tab. S1. (Cont.)

[a] N at 21, [b] N at 24, [c] $=CH_2$ at 4, [d] saturation at 3, [e] =O at 4, [f] O at 5, not 6, [g] O at 3, [h] extra methyl at 4, [i] N at 22, [j] N at 20, [k] saturation at 28, [l] indicates absence of double bond, [m] asymmetric bold bonds, [n] asymmetric hashed bonds, [o] asymmetric dashed bonds, [p] double bond at neighbouring C, [q] wavy double bond.
Compd. No. 1–23 [56]; Compd. No. 24–44, 69, 167, 168, 170 [57]; Compd. No. 45–65 [58]; Compd. No. 66–68, 70–75, 98–101 [59]; Compd. No. 76–97 [60]; Compd. No. 102–114 [61]; Compd. No. 115–141, 169 [62]; Compd. No. 142–166 [63].

Tab. S2. Observed and predicted activities (pK_i) of compounds

Cpd no.	Activity (pKi)			Cpd no.	Activity (pKi)		
	Obser-ved	Predicted			Obser-ved	Predicted	
		QSAR	Pharmacoph.			QSAR	Pharmacoph.
1[^,≠]	2.076	2.436	1.721	33[#,≠]	4.432	3.958	4.409
2[#,≠]	3.481	2.871	3.292	34[#,≠]	4.108	3.816	4.367
3[#,*]	4.022	3.290	4.260	35[#,ψ]	3.886	4.032	4.347
4[#,≠]	2.638	2.351	3.678	36[^,ψ]	4.131	3.616	4.155
5[^,ψ]	3.495	3.355	4.201	37[^,≠]	4.229	3.943	3.569
6[#,≠]	3.319	3.119	3.252	38[^,≠]	4.032	3.616	3.180
7[#,≠]	3.215	3.083	2.959	39[#,ψ]	4.319	4.092	4.398
8[$,≠]	3.959	3.083	3.509	40[#,*]	4.260	4.093	4.076
9[#,ψ]	3.538	3.042	4.137	41[^,ψ]	3.959	4.113	4.377
10[#,≠]	3.444	3.281	3.553	42[#,ψ]	4.469	4.072	4.538
11[#,ψ]	4.060	3.174	3.699	43[#,ψ]	4.301	4.047	4.125
12[#,ψ]	2.299	2.931	2.046	44[#,≠]	3.886	4.211	4.387
13[^,≠]	3.921	3.494	3.770	45[^,ψ]	3.444	3.227	2.824
14[#,≠]	3.456	3.136	3.328	46[$,ψ]	3.276	3.698	3.770
15[#,ψ]	2.577	2.891	2.536	47[#,≠]	4.260	3.692	4.004
16[#,≠]	2.194	2.845	2.222	48[#,ψ]	4.161	3.770	3.409
17[$,ψ]	2.416	2.885	1.824	49[$,≠]	4.367	3.724	4.409
18[$,ψ]	3.222	3.061	4.076	50[^,*]	3.959	3.839	4.337
19[#,≠]	2.785	2.875	1.824	51[#,*]	2.967	3.329	3.155
20[#,≠]	3.824	3.053	3.495	52[#,*]	3.056	3.351	3.086
21[#,*]	2.094	2.729	1.824	53[#,≠]	3.638	3.298	3.721
22[$,ψ]	2.614	2.913	2.688	54[#,≠]	2.876	3.314	3.097
23[#,≠]	3.180	3.177	4.046	55[#,ψ]	3.585	3.532	4.328
24[^,≠]	4.155	3.623	4.046	56[#,ψ]	3.469	3.921	4.357
25[#,*]	4.495	3.826	3.770	57[$,ψ]	4.387	3.937	3.658
26[$,ψ]	3.569	3.704	3.824	58[#,≠]	3.921	3.971	4.337
27[$,ψ]	4.229	3.641	3.796	59[^,ψ]	3.167	3.196	4.347
28[^,ψ]	3.620	3.462	3.387	60[$,≠]	3.745	3.805	3.125
29[$,*]	3.658	3.616	2.886	61[#,ψ]	3.770	3.379	4.387
30[#,*]	4.495	4.033	4.398	62[#,≠]	3.620	3.686	4.367
31[$,≠]	4.310	4.139	4.377	63[#,*]	4.060	3.544	4.387
32[^,≠]	3.569	3.475	3.337	64[#,≠]	3.409	3.860	3.886

Tab. S2. (Cont.)

Cpd no.	Activity (pKi)			Cpd no.	Activity (pKi)		
	Obser-ved	Predicted			Obser-ved	Predicted	
		QSAR	Pharmacoph.			QSAR	Pharmacoph.
65[#,≠]	2.860	3.201	4.201	106[#,≠]	3.260	2.955	4.347
66[#,≠]	2.936	3.667	3.357	107[#,≠]	3.060	2.935	3.721
67[#,ψ]	2.996	3.451	3.420	108[$,1]	3.886	3.332	4.276
68[#,≠]	2.609	3.236	3.420	109[#,ψ]	2.222	2.702	2.252
69[#,ψ]	2.097	1.970	2.432	110[^,*]	3.495	3.516	3.959
70[#,≠]	3.886	3.393	3.585	111[^,≠]	3.824	3.754	4.000
71[#,≠]	2.544	3.157	3.252	112[#,ψ]	2.504	2.461	2.602
72[$,ψ]	3.180	3.050	3.244	113[$,≠]	3.444	3.652	4.125
73[^,≠]	3.585	3.567	3.081	114[^,≠]	2.900	3.296	4.155
74[#,≠]	2.780	3.432	2.893	115[^,≠]	1.876	1.836	1.699
75[^,*]	3.276	3.340	3.319	116[#,≠]	2.854	2.422	1.602
76[^,≠]	4.180	3.367	4.229	117[^,≠]	2.907	2.773	2.854
77[^,≠]	4.215	3.294	3.824	118[#,≠]	1.754	2.496	1.721
78[#,≠]	3.260	3.637	3.886	119[#,≠]	2.620	2.626	1.745
79[$,*]	4.149	3.341	4.000	120[#,ψ]	2.538	2.179	1.745
80[#,≠]	3.824	3.766	4.268	121[^,≠]	2.951	2.646	2.398
81[^,*]	4.208	3.297	3.770	122[^,ψ]	1.735	2.283	1.602
82[$,≠]	4.260	3.693	4.201	123[#,≠]	1.947	2.457	1.620
83[#,ψ]	3.638	3.833	4.114	124[#,*]	0.449	2.099	1.602
84[$,ψ]	3.796	3.976	4.367	125[$,≠]	2.536	2.274	1.620
85[^,ψ]	3.721	3.779	4.420	126[$,ψ]	2.060	2.485	1.770
86[^,ψ]	3.420	3.556	4.319	127[#,≠]	1.316	1.674	1.602
87[#,≠]	4.229	3.606	4.004	128[#,≠]	0.731	1.576	1.699
88[^,ψ]	4.208	3.518	3.959	129[#,ψ]	1.996	1.526	1.745
89[#,ψ]	2.650	3.478	2.688	130[#,ψ]	1.348	1.887	1.620
90[^,ψ]	2.785	3.425	2.924	131[^,≠]	2.275	1.612	1.770
91[#,≠]	3.585	3.466	4.081	132[#,*]	1.695	1.884	1.678
92[#,≠]	3.194	3.762	4.237	133[#,*]	2.204	2.472	2.041
93[$,≠]	3.824	3.475	3.337	134[$,ψ]	2.886	2.540	1.602
94[^,ψ]	3.854	3.792	4.260	135[#,ψ]	2.244	2.569	1.959
95[#,ψ]	3.036	3.853	3.886	136[$,≠]	1.699	2.567	1.959
96[#,*]	4.377	3.059	4.292	137[#,≠]	2.810	2.521	2.004
97[#,≠]	3.921	3.479	4.444	138[#,ψ]	2.013	2.788	1.854
98[#,ψ]	4.081	3.453	3.886	139[#,ψ]	2.680	2.526	2.921
99[$,ψ]	3.201	3.623	4.000	140[#,≠]	2.114	2.233	1.538
100[#,ψ]	3.456	3.304	4.000	141[^,≠]	2.114	2.906	2.155
101[#,*]	3.310	3.052	3.886	142[#,ψ]	1.310	1.736	2.027
102[$,ψ]	3.678	3.154	4.194	143[#,ψ]	1.465	1.716	1.959
103[#,ψ]	3.310	2.935	3.770	144[#,ψ]	2.114	1.796	1.569
104[^,ψ]	3.252	3.407	3.959	145[#,≠]	1.807	1.962	1.585
105[#,≠]	3.456	3.106	4.056	146[$,≠]	2.509	1.945	1.921

Tab. S2. (Cont.)

Cpd no.	Activity (pKi)			Cpd no.	Activity (pKi)		
	Obser-ved	Predicted			Obser-ved	Predicted	
		QSAR	Pharmacoph.			QSAR	Pharmacoph.
147$^{\$,*}$	1.356	1.534	1.367	159$^{\#,\neq}$	1.662	2.617	1.569
148$^{\#,\neq}$	2.658	2.027	2.013	160$^{\wedge,\neq}$	2.469	1.800	1.699
149$^{\wedge,*}$	0.489	2.087	1.481	161$^{\wedge,\psi}$	1.561	1.719	1.337
150$^{\#,\neq}$	2.225	2.105	2.004	162$^{\wedge,\neq}$	1.342	1.996	2.018
151$^{\#,*}$	2.009	2.227	2.022	163$^{\#,*}$	2.149	2.957	1.745
152$^{\#,*}$	1.963	1.874	1.959	164$^{\$,\psi}$	2.161	2.158	1.658
153$^{\#,\psi}$	2.076	1.864	2.027	165$^{\wedge,*}$	2.678	2.377	2.022
154$^{\$,\neq}$	2.541	2.230	2.004	166$^{\$,\neq}$	2.076	1.968	1.959
155$^{\#,*}$	2.272	1.803	1.959	167$^{\wedge,\neq}$	3.456	3.189	3.387
156$^{\wedge,\neq}$	1.924	1.565	2.022	168$^{\#,\neq}$	4.469	3.474	4.495
157$^{\#,\neq}$	1.614	1.807	2.000	169$^{\#,\neq}$	3.959	2.770	3.678
158$^{\#,\neq}$	2.056	2.485	2.027	170$^{\#,\neq}$	3.728	3.251	3.824

$^{\#}$ QSAR Tr; $^{\$}$ QSAR Ts; $^{\wedge}$ QSAR Vs; * Pharmacophore Tr; $^{\psi}$ Pharmacophore Ts; $^{\neq}$ PharmacophoreVs

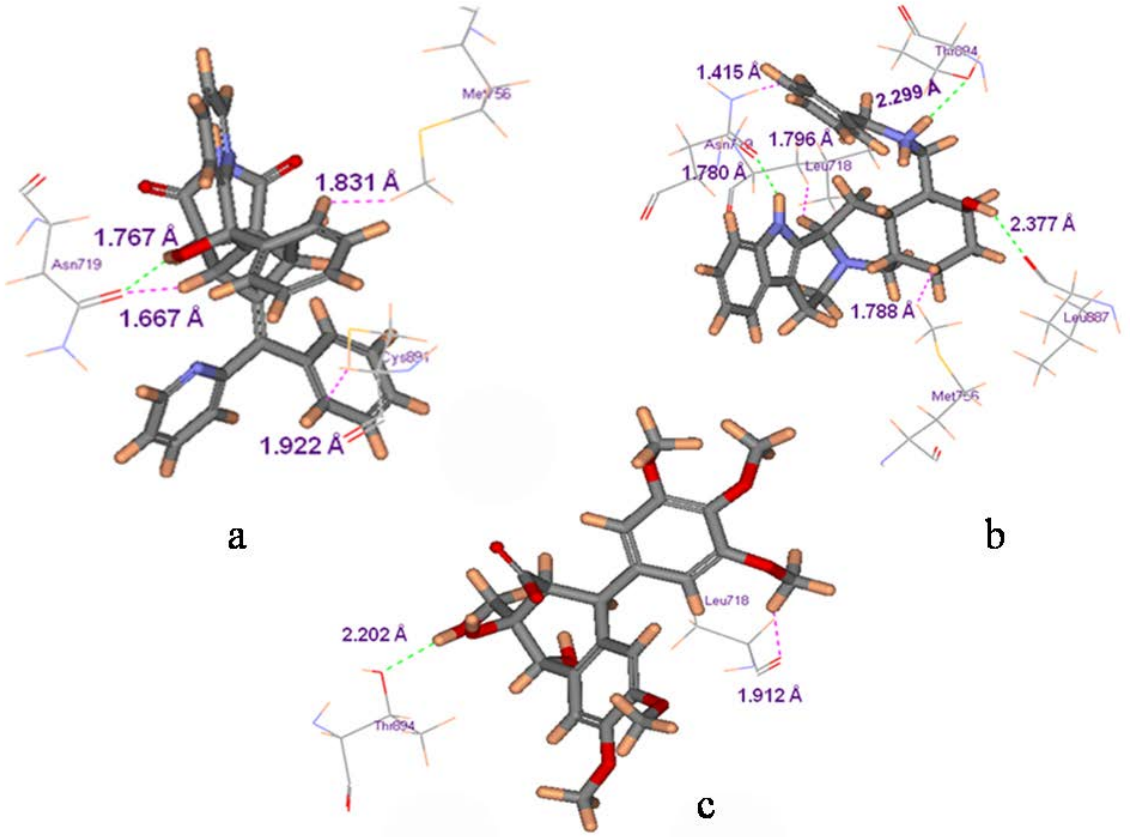

Fig. S1. Hit ligands at the binding site of 2OVH [64]. Catalytic residues are labeled.
(a) NCI0101316
(b) NCI0023681
(c) NCI0050131

References

[56] Zhl L, Tegley CM, Edwards JP, West SJ, Marschke KB, Gottardis MM, Mais DE, Jones TK.
5-Alkyl 1,2-dihydrochromeno[3,4-f]quinolines: A novel class of nonsteroidal progesterone receptor modulators.
Bioorg Med Chem Lett. 1998; 8: 3365–3370.
doi:10.1016/S0960-894X(98)00608-8

[57] Edwards JP, West SJ, Marschke KB, Mais DE, Gottardis MM, Jones TK.
5-Aryl-1,2-dihydro-5H-chromeno[3,4-f]quinolines as potent, orally active, nonsteroidal progesterone receptor agonists: The effect of D-ring substituents.
J Med Chem. 1998; 41: 303–310.
doi:10.1021/jm9705770

[58] Zhi L, Tegley CM, Kallel EA, Marschke KB, Mais DE, Gottardis MM, Jones TK.
5-Aryl-1,2-dihydrochromeno[3,4-f]quinolines: A novel class of nonsteroidal human progesterone receptor agonists.
J Med Chem. 1998; 41: 291–302.
doi:10.1021/jm9705768

[59] Zhi L, Tegley CM, Marschke KB, Mais DE, Jones TK.
5-Aryl-1,2,3,4-tetrahydrochromeno[3,4-f]quinolin-3-ones as a novel class of nonsteroidal progesterone receptor agonists: Effect of A-ring modification.
J Med Chem. 1999; 42: 1466–1472.
doi:10.1021/jm980723h

[60] Zhi L, Tegley CM, Pio B, Edwards JP, Motamedi M, Jones TK, Marschke KB, Mais DE, Risek B, Schrader WT.
5-Benzylidene-1,2-dihydrochromeno[3,4-f]quinolines as selective progesterone receptor modulators.
J Med Chem. 2003; 46: 4104–4112.
doi:10.1021/jm020477g

[61] Tegley CM, Zhi L, Marschke KB, Gottardis MM, Yang Q, Jones TK.
5-Benzylidene 1,2-dihydrochromeno[3,4-f]quinolines, a novel class of nonsteroidal human progesterone receptor agonists.
J Med Chem. 1998; 41: 4354–4359.
doi:10.1021/jm980366a

[62] Hamann LG, Winn DT, Pooley CF, Tegley CM, West SJ, Farmer LJ, Zhi L, Edwards JP, Marschke KB, Mais DE, Goldman ME, Jones TK.
Nonsteroidal progesterone receptor antagonists based on a conformationally-restricted, subseries of 6-aryl-1,2-dihydro-2,2,4-trimethylquinolines.
Bioorg Med Chem Lett. 1998; 8: 2731–2736.
doi:10.1016/S0960-894X(98)00482-X

[63] Hamann LG, Farmer LJ, Johnson MG, Bender SL, Mais DE, Wang MW, Crombie D, Goldman ME, Jones TK.
Synthesis and biological activity of novel nonsteroidal progesterone receptor antagonists based on cyclocymopol monomethyl ether.
J Med Chem. 1996; 39: 1778–1789.
doi:10.1021/jm950747d

[64] Madauss KP, Grygielko ET, Deng SJ, Sulpizio AC, Stanley TB, Wu C, Short SA, Thompson SK, Stewart EL, Laping NJ, Williams SP, Bray JD.
A structural and in vitro characterization of Asoprisnil: A selective progesterone receptor modulator.
Mol Endocrinol. 2007; 21: 1066–1081.
doi:10.1210/me.2006-0524
